# High Osmolarity Modulates Bacterial Cell Size through Reducing Initiation Volume in Escherichia coli

**DOI:** 10.1128/mSphere.00430-18

**Published:** 2018-10-24

**Authors:** Xiongfeng Dai, Manlu Zhu

**Affiliations:** a School of Life Sciences, Central China Normal University, Wuhan, China; University of Wyoming

**Keywords:** DnaA, cell cycle, cell size, hyperosmotic stress, initiation volume

## Abstract

Bacterial cell size is closely associated with biomass growth and cell cycle progression, including chromosome replication and cell division. It is generally proposed that Escherichia coli cells tightly control the timing of chromosome replication through maintaining a constant cell volume per origin upon initiating chromosome replication (constant initiation volume) under various growth conditions. Here, we quantitatively characterize the cell size and cell cycle of Escherichia coli cells growing exponentially under hyperosmotic stress, which is a common environmental stressor that profoundly affects the bacterial water content. The bacterial cell size is reduced by hyperosmotic stress, even though the C and D periods are remarkably prolonged, indicating a significantly reduced initiation volume. The reduced initiation volume originates from the higher concentration of DnaA initiator protein caused by water loss at high osmolarity. Our study shows suggests a fundamental role of water content in regulating bacterial cell size and has also revealed a new role of the DnaA protein in regulating the chromosome replication elongation beyond regulating the replication initiation process.

**IMPORTANCE** Bacterial cell size depends on growth rate, cell cycle progression, and the cell volume per origin upon initiating chromosome replication (initiation volume). Here, we perform the first systematic and quantitative study of the effect of hyperosmotic stress on the E. coli cell size and cell cycle. We find that hyperosmotic stress significantly reduces the initiation volume. The reduced initiation volume is attributed to the increased DnaA concentration caused by water loss at high osmolarity, indicating a fundamental role of water content in cell size and cell cycle regulation.

## Abstract (precis)

Bacterial cell size depends on growth rate, cell cycle progression, and the cell volume per origin upon initiating chromosome replication (initiation volume). Here, we perform the first systematic and quantitative study of the effect of hyperosmotic stress on the E. coli cell size and cell cycle. We find that hyperosmotic stress significantly reduces the initiation volume. The reduced initiation volume is attributed to the increased DnaA concentration caused by water loss at high osmolarity, indicating a fundamental role of water content in cell size and cell cycle regulation.

## INTRODUCTION

A quantitative understanding of the cell size regulation remains a fundamental challenge in biology. Bacteria manage to coordinate biomass growth with cell cycle progression to achieve size homeostasis (1–5). The C period (the period required for chromosome replication) and the D period (the period between the completion of chromosome replication and cell division) are two key stages of bacterial cell cycles (6, 7). The bacterial cell size is linked to growth rate and cell cycle progression by the following empirical equation:

(1) $$V=V_{i}\times 2^{\frac{C+D}{\tau }}$$

where *V* is the average cell size (volume) at division; *V_i_* is the average cell size per chromosome origin upon initiation of chromosome replication (referred to as “initiation volume” or unit cell), reflecting the timing of chromosome replication initiation; C+D is the sum of the C period and D period (the time between replication initiation and the completion of cell division); and τ is the mass doubling time (8–11). With the constancy of initiation volume and C+D period, Equation 1 quantitatively explains the intriguing positive exponential correlation between cell size and growth rate under different nutrient conditions (2, 3, 8, 9). Furthermore, recent phenomenological studies have confirmed that the cells initiate chromosome replication at a constant volume per origin under various growth perturbations and at the single-cell level (9–11). Those findings indicate the high robustness of Escherichia coli cells in controlling the timing of chromosome replication. With the constancy of initiation volume, bacterial cell size can be predicted given the information of growth rate and cell cycle parameters regardless of the actual growth conditions.

In previous studies, growth conditions used for studying cell size focused on extensive perturbations on biosynthetic pathways, including nutrient limitation, DNA replication limitation, transcription inhibition, translation inhibition, and perturbation of fatty acid synthesis and cell wall synthesis (5, 9, 12, 13). In their natural habitat, bacterial cells frequently confront abruptly changing solute concentrations, which result in nonoptimal osmolarity (14). For example, E. coli encounters hyperosmotic stress when infecting the urinary tract due to the high osmolarity of the bladder and urine (15–17). The high salt contained in a Western diet has also been found to significantly affect the physiology of the gut microbiome, further accelerating the emergence of various human disorders (18). The effect of hyperosmotic stress is highly attractive since it profoundly affects the cellular water content, which is the basis of life (19–21). Water content accounts for the majority of the bacterial cellular mass and is the predominant factor that sets the total cellular volume of bacterial cells (19, 20). Under hyperosmotic stress, the rapid loss of both water and turgor pressure severely inhibits bacterial biomass growth (20). To cope with this severe physiological challenge, bacterial cells must initiate related stress response programs to accumulate osmolytes, such as potassium, glutamate, and trehalose, to reestablish the balance of external and internal osmolarity (22). However, this process only partially restores the water content and growth of bacteria (19, 23).

Though hyperosmotic stress profoundly affects bacteria physiology through reducing the cellular water content, it remains unclear how it affects the cell size and cell cycle of bacteria. In this study, we quantitatively characterized the cell size and cell cycle progression of E. coli growing under hyperosmotic stress. We found that initiation volume strongly decreased under hyperosmotic stress, implying an important role of water in cell size regulation.

## RESULTS

### Cell size and cell cycle parameters under hyperosmotic stress.

**FIG 1:**
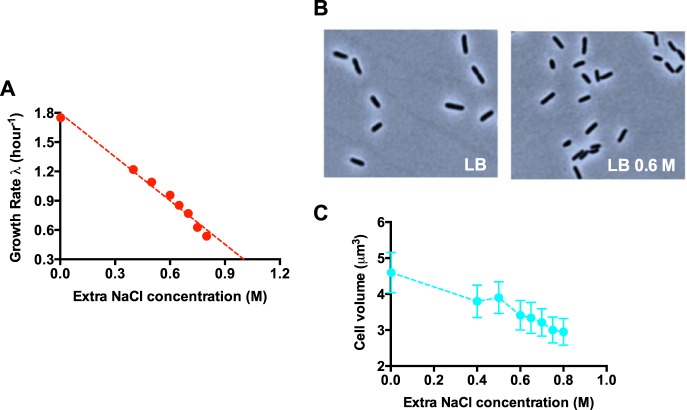
Cell size and growth rate of *E. coli* under hyperosmotic stress. (A) Growth rate of *E. coli* in different osmolarities. The original LB medium (containing 1% NaCl) is supplemented with different concentrations of extra NaCl. (B) Phase-contrast images of *E. coli* at different osmolarities. (C) Average cell volume of *E. coli* at different osmolarities.

We focused on the wild-type E. coli K-12 MG1655 strain exponentially growing under hyperosmotic stress created by large amounts of sodium chloride (NaCl) (19). The growth rate of E. coli in LB medium decreased 70% from 0 M extra NaCl to 0.8 M extra NaCl (Fig. 1A). The cell size of E. coli in various osmolarities was then measured using phase-contrast microscopy and the ImageJ software (Fig. 1B). The cell size of E. coli steadily decreases at high osmolarity (Fig. 1C). A similar result was also obtained for cells growing in glucose medium (see Fig. S1A and B in the supplemental material).

10.1128/mSphere.00430-18.1 FIG S1 Growth rate, cell size, and cell cycle parameters of E. coli growing in glucose medium with different osmolarities. (A) Growth rate. (B) Cell size. (C) C period; (D) D period. Data are average of the results from triplicates, with standard deviations being within 5% to 10%. (E) C+D period. (F) Initiation volume. Download FIG S1, PDF file, 0.03 MB. Copyright © 2018 Dai and Zhu. 2018 Dai and Zhu This content is distributed under the terms of the Creative Commons Attribution 4.0 International license.

**FIG 2:**
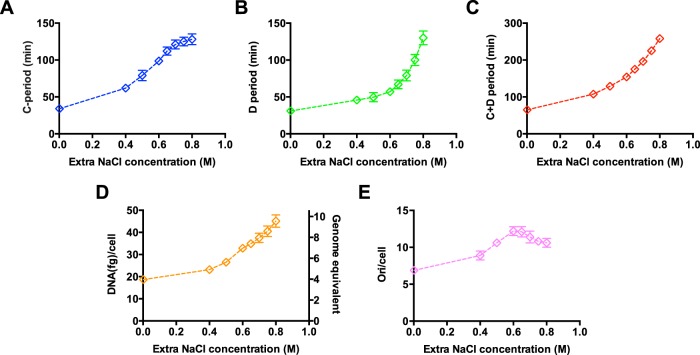
Cell cycle parameters and DNA content of *E. coli* under hyperosmotic stress. Cells were grown in LB medium supplemented with different levels of sodium chloride as in Fig. 1. (A) C period of *E. coli* at different osmolarities. (B) D period of *E. coli* at different osmolarities. (C) C+D period of *E. coli* at different osmolarities. (D) Cellular DNA content of *E. coli* at different osmolarities. (E) Chromosome origins per cell of *E. coli* at different osmolarities. The chromosome origin/cell equals 2^(C+D)/τ^. Data are average of the results from triplicates, with standard deviations being within 5% to 10%.

We next characterized related cell cycle parameters of E. coli growing under hyperosmotic stress. The C period was measured by the *ori*-*ter* method using quantitative PCR (qPCR). Strikingly, the C period of E. coli growing in LB medium increases by almost 4-fold (from 34 min to 128 min) from 0 M extra NaCl to 0.8 M extra NaCl, indicating that the movement of replication forks greatly slowed down under hyperosmotic stress (Fig. 2A). This result for the C period was further confirmed by the DNA increment method (Fig. S2). Hyperosmotic stress also causes a 4-fold increase in the D period (Fig. 2B). The increase in both the C and D periods was also found for cells growing in glucose medium (Fig. S1C to E). Overall, the time from chromosome replication initiation to the completion of cell division (C+D period) was severely prolonged by hyperosmotic stress (Fig. 2C). The increased C+D period further led to an increase in both the cellular DNA content per cell and chromosome origins per cell [equal to 2^(C+D)/τ^] (Fig. 2D and E).

10.1128/mSphere.00430-18.2 FIG S2 C period of E. coli in hyperosmotic stress (LB medium) obtained by DNA increment method. In the text, the C period was mainly measured by the qPCR method. To further confirm the data of the C period, we applied a second method, a DNA increment method, to measure it. The detailed procedure is described by Bipatnath et al. (42) and Churchward and Bremer (41). The basic principle is that at time zero, a large amount of chloramphenicol (200 µg/ml) or rifampin (200 µg/ml) was added into the bacterial culture so that initiation of new rounds of chromosome replication was blocked. However, after the block of replication initiation, the ongoing rounds of replications will continue until finishing so that the total DNA amount of the culture will continue to increase until the time (Tc) when all the ongoing replication forks complete DNA chain elongation. The time Tc corresponds to the C period. The relative change of DNA amount after time Tc corresponds to the *oriC*/genome equivalent. We measured the relative DNA accumulation curve (taking the data point at time zero as 1) after blocking chromosome replication initiation for cells growing in LB medium supplemented with different concentrations of sodium chloride. From the data described above, it is clear that C period increases strongly at high osmolarity. The C period values here are 38 min (LB), 62 min (LB plus 0.4 M NaCl), 82 min (LB plus 0.5 M NaCl), and 101 min (LB plus 0.6 M NaCl), respectively. This result is consistent with the result shown in Fig. 2A obtained by qPCR method. Download FIG S2, PDF file, 0.05 MB. Copyright © 2018 Dai and Zhu. 2018 Dai and Zhu This content is distributed under the terms of the Creative Commons Attribution 4.0 International license.

### Decreased initiation volume under hyperosmotic stress.

**FIG 3:**
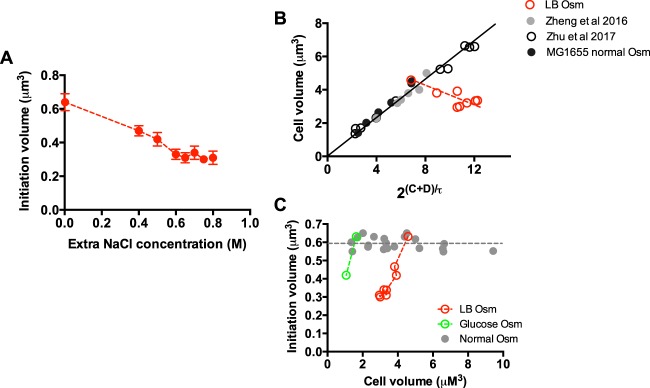
Initiation volume of *E. coli* under hyperosmotic stress. (A) Initiation volume of *E. coli* growing in LB medium with different osmolarities (Osm). Data are average of the results from triplicates, with standard deviations being within 5% to 10%. (B) Correlation between the cell size and 2^(C+D)/τ^ in normal osmolarity and high osmolarity. Data points of LB medium with different osmolarities (red points) correspond to data in Fig. 1C and 2E. The data points in normal osmolarity originate from Fig. S3, a study by Zheng et al. (11), and a study by Zhu et al. (13), and fit with a linear line. For the study by Zhu et al., the strain used was wild-type K-12 NCM3722. Data points of the NCM3722 strain are plotted together with that of the MG1655 strain because of their similar initiation volumes found at normal osmolarity, ∼0.6 µM^3^. (C) Correlation between the initiation volume and cell size at normal osmolarity and high osmolarity. Data points at normal osmolarity originate from Fig. S3, a study by Zheng et al., and a study by Zhu et al. Data points of high osmolarity in both LB medium and glucose medium correspond to data in Fig. 1C, 3A, and S1B and F.

Based on the prediction of Equation 1, the increased 2^(C+D)/τ^ should in principle cause a remarkable increase of cell size under hyperosmotic stress if the initiation volume remains unchanged. However, cell size actually decreases under hyperosmotic stress (Fig. 1C). This result suggests that hyperosmotic stress causes a significant reduction in initiation volume. As shown in Fig. 3A, the initiation volume of cells decreases 50% from 0 M extra NaCl to 0.6 M extra NaCl, further reaching a lower bound at even higher NaCl concentrations. The decrease of initiation volume at high osmolarity has also been observed for cells growing in glucose medium (Fig. S1F). The decrease in initiation volume could be clearly judged from the correlation between cell size and2^(C+D)/τ^. In normal osmolarity, the correlation between cell size and 2^(C+D)/τ^ is linear, indicating a constant initiation volume (black and gray symbols in Fig. 3B) (9, 11, 13). However, this linear correlation was remarkably broken under hyperosmotic stress (red symbols in Fig. 3B). We further plotted the initiation volume versus cell size as another way to see the constancy of initiation volume at normal osmolarity, as well as its decrease at high osmolarity (Fig. 3C and S3).

10.1128/mSphere.00430-18.3 FIG S3 Cell size and cell cycle parameters of MG1655 strain under different nutrient conditions (normal osmolarity). Growth conditions include LB medium (λ = 1.78/h), rich defined medium (RDM) plus glucose medium (λ = 1.71/h), RDM plus glycerol medium (λ = 1.46/h), glucose plus Casamino Acids (cAA) medium (λ = 1.2/h), glycerol plus Casamino Acids medium (λ = 0.87/h), and glucose minimal medium (λ = 0.7/h). (A) C period. (B) D period. (C) Origins per cell. (D) Cell size. (E) Initiation volume. Download FIG S3, PDF file, 0.04 MB. Copyright © 2018 Dai and Zhu. 2018 Dai and Zhu This content is distributed under the terms of the Creative Commons Attribution 4.0 International license.

### Increased DnaA concentration under hyperosmotic stress.

**FIG 4:**
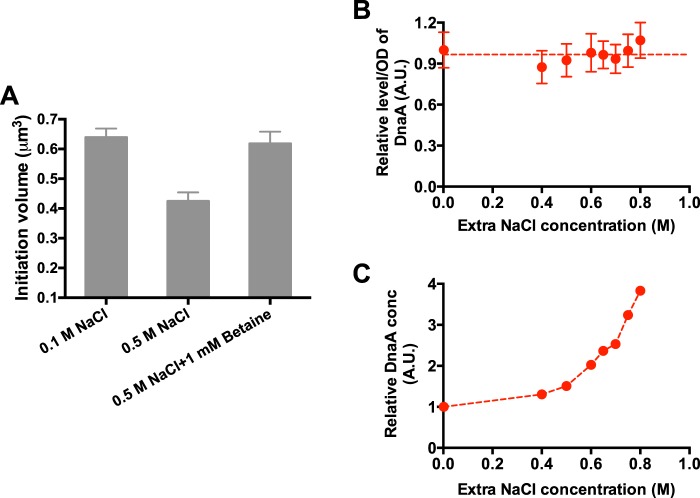
DnaA concentrations at different osmolarities. (A) Initiation volume of *E. coli* growing in glucose medium of normal osmolarity (0.1 M NaCl), high osmolarity (0.5 M NaCl), and high osmolarity supplemented with 1 mM betaine. (B) DnaA content/OD_600_ at LB medium supplemented with different osmolarities. Data are average of the results from triplicates, with standard deviations being within 15%. A.U., absorbance units. (C) Relative DnaA concentration (conc) at LB medium of different osmolarities. It corresponds to DnaA content (per OD quantity) normalized by the water content (Fig. S5F).

One of the major physiological differences between cells growing in normal osmolarity and those growing at high osmolarity is the cellular water content. We added betaine, a strong osmoprotectant, to the high-osmolarity medium to restore the water content of E. coli (19). Since normal LB medium consists of betaine (24), we managed to perform the study in the morpholinepropanesulfonic acid (MOPS) glucose medium. As shown in Fig. 4A, the addition of betaine restored the initiation volume of cells growing at high osmolarity. Moreover, the cell growth, cell size, and cell cycle parameters were also restored by betaine (Fig. S4). Those results indicate a potential role of water in regulating bacterial cell size.

10.1128/mSphere.00430-18.4 FIG S4 Growth rate, cell size, and cell cycle parameters of E. coli at high osmolarity supplemented with osmoprotectant, betaine. (A) Growth rate. (B) Cell size. (C) C period. (D) D period. Data are the average from the results of triplicates, with standard deviations being within 10%。 Download FIG S4, PDF file, 0.03 MB. Copyright © 2018 Dai and Zhu. 2018 Dai and Zhu This content is distributed under the terms of the Creative Commons Attribution 4.0 International license.

10.1128/mSphere.00430-18.5 FIG S5 Relative water content of E. coli at different osmolarities. The product of the cell size and cell number/OD is used as a proxy of the relative water content of E. coli at different osmolarities. (A and B) The growth condition is glucose minimal medium supplemented with different concentrations of sodium chloride. The red triangle corresponds to data point of glucose plus 0.5 M NaCl medium supplemented with 1 mM glycine betaine. The cell number/OD and cell size of E. coli both decreases with growth rate in glucose medium with higher osmolarity (A), indicating lower water content at high osmolarity. Our way of estimating water content is quantitatively consistent with the data from water content determinations in a study by Cayley and Record (19), who also used the MG1655 strain and the same glucose minimal medium (purple triangles in panel B). The water content of E. coli in glucose minimal medium supplemented with 0.1 M NaCl (normal osmolarity) is set as “1.” (C and D) Water estimation of E. coli under different nutrient conditions at normal osmolarity. At normal osmolarity, the cell size decreases, while the cell number/OD increases under nutrient limitation (C), demonstrating constant water content (D). (E and F) Data from E. coli in LB medium. The open circles symbols show data of wild-type cells at different osmolarities. The open triangles show data of tCRISPR-*dnaA* strain (Fig. 6 of main text) at two osmolarities (LB medium and LB plus 0.6 M medium), where data points of LB plus 0.6 M medium include values at all the arabinose concentrations used in this study. Download FIG S5, PDF file, 0.1 MB. Copyright © 2018 Dai and Zhu. 2018 Dai and Zhu This content is distributed under the terms of the Creative Commons Attribution 4.0 International license.

The decreased initiation volume suggests acceleration in the process of chromosome replication initiation at high osmolarity. One possibility is that the cellular concentration of the DnaA protein, the master regulator of chromosome replication initiation of E. coli, increases at high osmolarity. Previous studies have demonstrated that alterations in the DnaA levels could perturb the timing of the chromosome initiation process of E. coli (9, 25–27). To test the above-mentioned hypothesis, we measured the DnaA protein content using mass spectrometry. The DnaA content per optical density at 600 nm (OD_600_) remains almost constant at different osmolarities (Fig. 4B). To obtain the DnaA concentration, we further deduced the relative water content of cells at different osmolarities. The product of the cell size and cell number per OD was used as the proxy of the water content (Fig. S5). As shown in Fig. S5B and F, the water content of E. coli decreases dramatically at high osmolarity, consistent with previous findings (19, 21). Therefore, the relative DnaA concentration corresponds to the DnaA content per OD_600_ normalized by the water content. As shown in Fig. 4C, the cellular DnaA concentration dramatically increases with higher osmolarity.

### Effects of DnaA overexpression and DnaA knockdown on initiation volume.

**FIG 5:**
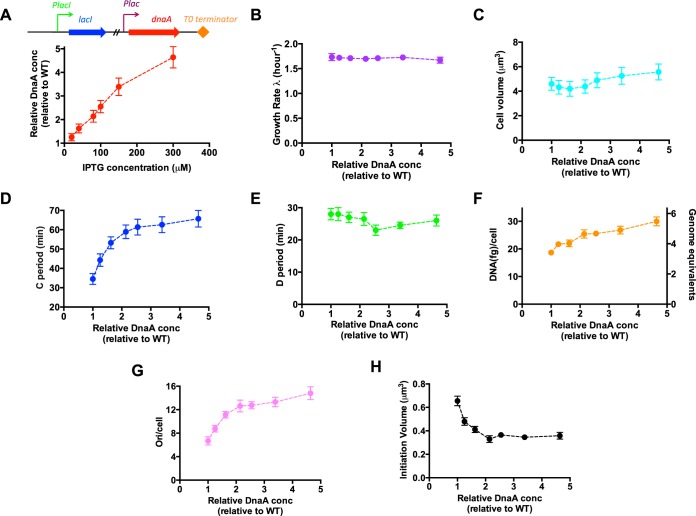
Cell size and cell cycle parameters of *E. coli* strain overexpressing DnaA protein at normal osmolarity. Cells were grown in LB medium. (A) Relative DnaA concentrations at different IPTG inducer levels. The DnaA concentration of wild-type cells is set as “1”. The expression of DnaA is driven by the P*lac* promoter, which is under the regulation of the LacI repressor. (B) Growth rate of *E. coli* upon DnaA overexpression. (C) Cell size of *E. coli* upon DnaA overexpression. (D) C period of *E. coli* upon DnaA overexpression. (E) D period of *E. coli* upon DnaA overexpression. (F) Average DNA content per cell of *E. coli* upon DnaA overexpression. (G) Origins per cell of *E. coli* upon DnaA overexpression. (H) Initiation volume of *E. coli* upon DnaA overexpression. Data are average of the results from triplicates, with standard deviations being within 5% to 10%.

We next investigated the effect of artificial perturbation of DnaA expression on the bacterial cell size and cell cycle. We first studied the case of DnaA overexpression at normal osmolarity. The DnaA overexpression strain, FL68, harbors an isopropyl thio-β-d-galactopyranoside (IPTG)-inducible P*lac*-*dnaA* plasmid (Fig. 5A). Varying the concentrations of IPTG inducers enables various degrees of DnaA overexpression. The dynamic range of DnaA overexpression is 5-fold at normal osmolarity (Fig. 5A). Upon DnaA overexpression, the growth rate remains invariant (Fig. 5B), while the cell size increased slightly (Fig. 5C). Strikingly, the C period increases strongly upon DnaA overexpression, being similar to the case of hyperosmotic stress (Fig. 5D). This indicates that DnaA can also affect the process of replication elongation. Instead, the D period is largely independent of the DnaA concentrations (Fig. 5E). The invariance of growth rate and delayed cell cycle (C+D) with DnaA overexpression further lead to a 1.5-fold increase in the DNA content per cell and a >2-fold increase in the origins per cell (Fig. 5F and G). The origins per cell increase strongly while the cell size changes only slightly upon DnaA overexpression, indicating a remarkable reduction in initiation volume. As shown in Fig. 5H, the initiation volume indeed significantly decreases under low degrees of DnaA overexpression, consistent with previous findings (26, 27). Moreover, initiation volume reaches a lower bound at higher DnaA concentrations, similar to the finding under hyperosmotic stress (Fig. 3A).

**FIG 6:**
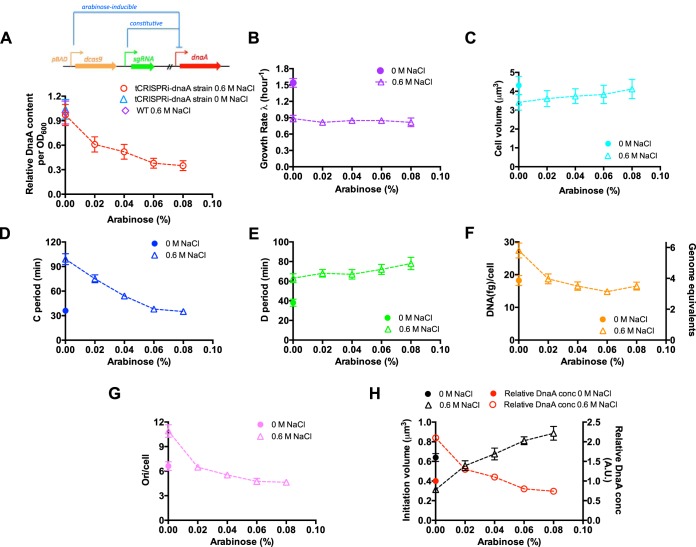
Cell size and cell cycle parameters of the *E. coli* tCRISPRi**-**DnaA strain at a fixed high osmolarity. Cells were grown at a fixed high osmolarity (LB plus 0.6 M NaCl medium). The expression of DnaA protein is repressed by the CRISPR-dcas9 system in an arabinose-dose-dependent manner. (A) Relative DnaA content (per OD_600_ quantity) of the tCRISPRi-DnaA strain growing in LB plus 0.6 M NaCl medium supplemented with different levels of arabinose (red circles). The blue triangle corresponds the relative DnaA content of the tCRISPRi-DnaA strain growing in normal LB medium. The DnaA content of wild-type cells at LB plus 0.6 M NaCl (purple symbol) is set as “1”. (B) Growth rate of *E. coli* at high osmolarity upon DnaA knockdown (purple open triangles). (C) Cell size of *E. coli* at high osmolarity upon DnaA knockdown (cyan open triangles). (D) C period of *E. coli* at high osmolarity upon DnaA knockdown (blue open triangles). (E) D period of *E. coli* at high osmolarity upon DnaA knockdown (green open triangles). (F) Average DNA amount per cell of *E. coli* at high osmolarity upon DnaA knockdown (orange open triangles). (G) Origins per cell of *E. coli* at high osmolarity upon DnaA knockdown (pink open triangles). (H) Initiation volume (black triangles) of *E. coli* at high osmolarity upon DnaA knockdown. The relative DnaA concentration (red circles) corresponds to DnaA content per OD (panel A) normalized by the water content (triangles in Fig. S5F). Data are average of the results from triplicates, with standard deviations being ∼10%.

If DnaA is indeed the limiting factor of initiation volume under hyperosmotic stress, artificial knockdown of DnaA expression is expected to restore the initiation volume. To test this hypothesis, we applied CRISPR-dcas9 system to repress the expression of DnaA protein using a tunable CRISPR interference (tCRISPRi)-*dnaA* strain (Fig. 6A) (28). The expression of DnaA protein (per OD_600_ quantity) is repressed by the CRISPR-dcas9 system in an arabinose-dose-dependent manner at a fixed high osmolarity (LB plus 0.6 M NaCl). From 0% to 0.08% arabinose, the repression of DnaA expression is ∼3-fold (red symbols in Fig. 6A). Moreover, in the absence of arabinose, the DnaA abundances (per OD quantity) of the tCRISPRi-*dnaA* strain at both normal osmolarity and high osmolarity are similar to those of wild-type cells (blue triangle and purple diamond in Fig. 6A). We next investigated related cell cycle parameters of the tCRISPRi-*dnaA* strain growing at a fixed high osmolarity (LB plus 0.6 M NaCl medium). The growth rate of E. coli cells remains constant while cell size increases slightly upon DnaA knockdown. Strikingly, DnaA knockdown at high osmolarity completely restores the C period (99 min) to its value at normal osmolarity (∼35 min) (Fig. 4D). This finding is consistent with the finding in DnaA overexpression study that DnaA inhibits the chromosome replication elongation. In contrast, the D period increases only slightly upon DnaA knockdown (Fig. 6E). The invariance of the growth rate and shortened C+D period upon DnaA knockdown further lead to a 1.5-fold decrease in the DNA content per cell and a 2.5-fold decrease in the origins per cell (Fig. 6F and G). These measurements of cell cycle parameters allowed us to deduce the effect of DnaA knockdown on initiation volume. Under zero-arabinose conditions, the initiation volume of the tCRISPRi-*dnaA* strain at normal osmolarity (in LB medium) is half of what it is at high osmolarity (in LB plus 0.6 M NaCl medium) (Fig. 6H), the same as the case of wild-type cells (Fig. 3A). Strikingly, DnaA knockdown increases the initiation volume of E. coli by nearly 3-fold at high osmolarity. The initiation volume even exceeds the value of wild-type cells at normal osmolarity (∼0.6 μM^3^), reaching 0.85 μM^3^ at 0.08% arabinose. As a reference, we plotted the DnaA concentration (DnaA per OD normalized by water content, as shown in Fig. S5F) together with initiation volume (Fig. 6H). The plot clearly shows that initiation volume remarkably increases with decreasing level of DnaA.

**FIG 7:**
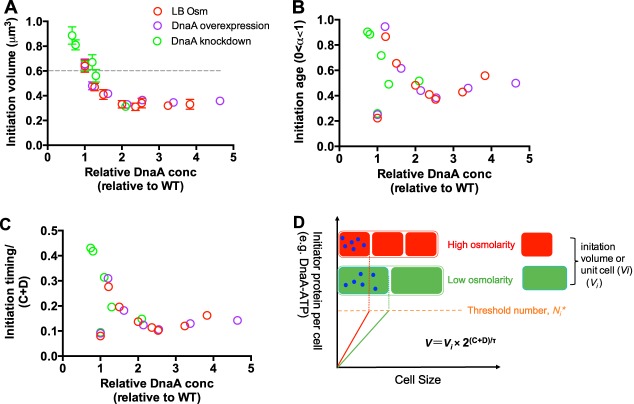
Quantitative correlation of initiation volume, initiation age, and initiation timing/(C+D) period with the DnaA concentration. (A) Correlation between the initiation volume and DnaA concentration under three conditions. Data points of hyperosmotic stress in LB medium are from Fig. 3A and 4C. Data points of DnaA overexpression are the same as those in Fig. 5H. Data points of DnaA knockdown at high osmolarity (LB plus 0.6 M NaCl) are the same as those in Fig. 6H.The DnaA concentration of wild-type cells growing in normal LB medium is set as “1”. The gray dashed line denotes the initiation volume at normal osmolarity. Data are average of the results from triplicates, with standard deviations being ∼10%. (B) Correlation between the initiation age and DnaA concentration under three conditions. (C) Correlation between initiation timing/(C+D) period and DnaA concentration under three conditions. (D) The threshold initiation model.

Finally, we analyzed the quantitative correlation between the initiation volume and DnaA concentration under hyperosmotic stress, DnaA overexpression, and DnaA knockdown. Strikingly, the correlation of initiation volume versus DnaA under those three independent conditions collapses into the same curve (Fig. 7A), demonstrating an important role of DnaA concentration in setting the initiation volume. We also find that the correlation of initiation age and initiation timing versus (C+D) period with DnaA concentration (Fig. 7B and C) under three conditions largely overlap each other. The above-mentioned results strongly support the idea that the increased DnaA concentration underlies the decreased initiation volume of E. coli upon hyperosmotic stress.

## DISCUSSION

The concept of the constant initiation volume (or initiation mass), as described in Equation 1, was originally proposed by Donachie in 1968 to explain the exponential correlation between cell size and growth rate of bacteria under nutrient limitation (8, 29, 30). Donachie deduced the constant initiation volume model through combining the cell size data of Salmonella enterica serovar Typhimurium and the Cooper-Helmstetter model of cell cycle (29, 30). Recent extensive quantitative studies have confirmed the constancy of initiation mass in various modes of growth limitation and at single-cell levels (9–11). In this study, we find that the initiation volume for E. coli is reduced by hyperosmotic stress, a stress condition that profoundly affects the cellular water content. The reduced initiation volume is attributed to the increased DnaA concentration caused by water loss at high osmolarity, indicating a fundamental role of water content in cell size and cell cycle regulation.

It should be noted that at normal osmolarity, the term of “initiation volume” is interchangeable with “initiation mass” due to the constancy of cell mass density (constant water content) (12, 31). Donachie first used the term initiation mass (8) since the cell size data he used originated from Schaechter et al. (30), who took optical cell density/cell (cell mass) to represent cell size (6). However, in the case of changing osmolarity, initiation volume is no longer strictly proportional to initiation mass because of the altered dry mass density. To investigate this issue, we measured the dry mass/OD_600_ of E. coli cells growing at different osmolarities. Dry mass/OD_600_ remains nearly constant at different osmolarities (black circles in Fig. S6A); therefore, the dry mass density (normalized by water content) strongly increases at high osmolarity (red circles in Fig. S6A). In this case, the initiation mass (the product of initiation volume and dry mass density) still remains constant at different osmolarities (black diamond in Fig. S6) (9), confirming that the theory of constant initiation mass holds true even if cell size changes due to altered water content.

10.1128/mSphere.00430-18.6 FIG S6 Initiation mass of E. coli under hyperosmotic stress. At normal osmolarity, the terms “initiation mass” and “initiation volume” are interchangeable because of the constant cell mass density (Basan et al. [12] and Nanninga and Woldringh [31]). However, the relative dry mass density increases because of water loss at high osmolarity. (A) The dry mass/OD_600_, relative water content, and relative dry mass density of E. coli cells growing in LB medium at different osmolarities (from 0 M extra NaCl to 0.6 M extra NaCl). The relative dry mass density corresponds to dry mass/OD normalized by the relative water amount. The relative water amount and relative dry mass density at normal osmolarity are set as “1.” (B) Initiation mass of E. coli at different osmolarities. The initiation mass (black symbols) corresponds to the product of initiation volume (Fig. 3A) and relative dry mass density (A). The data point of initiation dry mass at normal osmolarity is set as “1.” Initiation volume (orange symbols) corresponds to the data of Fig. 3A. Download FIG S6, PDF file, 0.04 MB. Copyright © 2018 Dai and Zhu. 2018 Dai and Zhu This content is distributed under the terms of the Creative Commons Attribution 4.0 International license.

Our study combining hyperosmotic stress, DnaA overexpression, and DnaA knockdown firmly establishes the quantitative correlation between initiation volume and DnaA (Fig. 7A), in which the initiation volume can be modulated at a range of 3-fold by perturbing the DnaA concentrations. Those results clarify the important role of DnaA in controlling initiation volume and support the threshold initiation model regarding the regulation of the timing of chromosome replication initiation (9, 32, 33). In the threshold initiation model (Fig. 7D), E. coli cells reach initiation volume when they have accumulated a fixed threshold number (N_i_*) of chromosome replication initiators (e.g., the ATP form of DnaA). Furthermore, in normal osmolarity, the DnaA concentration (*c_dna_*), is supposed to remain constant under different growth conditions, leading to the constant initiation volume (mass) as found in previous studies (9, 34–36). However, in the case of high osmolarity, *c_dna_* increases due to water loss. Therefore, the accumulation speed of replication initiators per cell in high osmolarity (red line in Fig. 7D) is faster than it is in normal osmolarity (green line in Fig. 7D). In this case, N_i_* could be reached at a smaller initiation volume in high osmolarity (red rectangle in Fig. 7D) than it could in normal osmolarity (green rectangle in Fig. 7D). The reduced initiation volume ultimately leads to a smaller cell size of E. coli at high osmolarity.

Our study has also found that hyperosmotic stress significantly prolongs the C and D periods (Fig. 2A to C). The prolonged C period has also been observed in the case of DnaA overexpression (Fig. 5D). Moreover, knockdown of DnaA expression at high osmolarity completely restores the C period to normal value (Fig. 6D), indicating that the increased DnaA concentration plays a major role in prolonging C period at high osmolarity. This finding suggests a new role of the DnaA protein in regulating the chromosome replication elongation process beyond regulating the replication initiation. One possibility is that alteration of the DnaA protein level significantly perturbs the intracellular dinucleoside triphosphate (dNTP) substrate pools, further affecting the replication elongation rate. This effect could be achieved if DnaA directly regulates the expression of ribonucleotide reductase (RNR), the rate-limiting enzyme in dNTP synthesis, as suggested by previous studies (13, 37). Another possibility is that DnaA directly binds to the chromosome, further interfering with the replication elongation process. Hyperosmotic stress also causes an increased D period, suggesting its inhibitory effect on the cell division process. Previous studies have shown that titration of cell division-related protein, such as FtsZ or MreB, could significantly perturb the D period (9, 11). In light of this, it is possible that the expression of related protein decreases at high osmolarity. It is also possible that the increased intracellular osmolyte pools (potassium and glutamate) at high osmolarity have a direct inhibitory effect on the cell division process.

## MATERIALS AND METHODS

### Strains and growth medium.

The strains used in this study include the E. coli wild-type K-12 MG1655 strain, its DnaA overexpression strain derivative, the FL68 strain, as well as the its DnaA knockdown derivative, the tCRISPRi-DnaA strain. The media used in this study are routine LB medium and MOPS-buffered glucose minimal medium (Coolaber Beijing) as described by Dai et al. (38, 39) and Basan et al. (12). Different concentrations of sodium chloride (NaCl) were supplemented to the medium to adjust the osmolarity.

To construct a DnaA overexpression strain, the coding sequence of *dnaA* was directly fused with P*lac* promoter through overlapping PCR (Green golden PCR mix; Tsingke Biotech, China). The fused P*lac-dnaA* cassette was inserted into the HindIII/SpeI sites of the pBBR1-MCS plasmid, resulting in the pBBR1-*dnaA* vector. The P*lacI*-*lacI* cassette was PCR amplified using the MG1655 genome as the template and inserted into the KpnI/HindIII sites of pBBR1-*dnaA* to obtain the pBBR-*lacI*-P*lac*-*dnaA* vector. The pBBR-*lacI*-P*lac*-*dnaA* vector was then transformed into the wild-type MG1655 strain, generating the FL68 strain.

The tCRISPRi-DnaA strain (MG1655 background) used for DnaA knockdown study was kindly provided by Suckjoon Jun and Fangwei Si (UCSD). The detailed information of this strain is described in studies by Si et al. (9) and Li et al. (28).

### Cell growth.

Cell growth was performed in a 37°C air bath shaker (220 rpm). A standard cell growth procedure contains three steps, seed culture, preculture, and experimental culture. Cells from a fresh colony in the LB plate were inoculated into LB broth plus 0.3 M NaCl medium (for early adaptation to the high osmolarity) and grown for several hours as seed culture. Seed cultures were then transferred into the medium of the final experimental culture (e.g., high-osmolarity medium) and grown overnight at 37°C as preculture. In the next day, the overnight precultures were inoculated into the same medium as for the preculture at an initial OD_600_ of ∼0.015 as experimental culture. For each condition, we took at least six OD_600_ points (range, 0.05 to 0.5) to obtain an exponential-growth curve for the calculation of growth rate. The OD_600_ was measured using a Thermo Scientific Genesys 30 spectrophotometer.

### Cell size measurement.

Five microliters of cell culture at an OD_600_ of ∼0.3 was applied to a slide glass covered with a thin layer of agar (the agar immobilizes the cells). Cells were imaged using phase-contrast mode of a Nikon Eclipse Ti-80 microscope. For each condition, the images of 500 to 1,000 individual cells were taken. Cell length (*L*) and width (*W*) were extracted using the ImageJ software. The cell volume (*V*) was calculated using the equation $V=\pi W^{2}/4\times (L-\frac{W}{3}).$

### Measurement of cell cycle parameters and DNA content.

The C period is measured by two methods. The first method is measuring the *ori*-to-*ter* ratio by qPCR. The total genomic DNA of E. coli was extracted using a bacterial total genome DNA extraction kit (Tiangen Biotech Co., Ltd.). The primers (Tsingke Biotech Co.) used for amplifying the DNA region proximal to the origin (*oriC*) and terminus (*ter*) are 3923874fw (5′-GCCCTGTGGATAACAAGGAT-3′) and 3923874rv (5′-CCTCATTCTGATCCCAGCTT-3′) for *oriC*, and ter-fw (5′-TCCTCG CTGTTTGTCATCTT) and ter-rv (5′-GGTCTTGCTCGAATCCCTT) for *ter*. The qPCR reactions were performed using a SYBR green premix (Yeasen Biotech, Shanghai, China), according to the instruction manual. The qPCR reaction was carried out in a Bio-Rad CFX96 Touch real-time PCR system with the following procedures: 95°C for 15 min, followed by 40 cycles of 95°C for 30 s, 60°C for 30 s, and 72°C for 30 s. Calculation of the C period is based on *ori/ter*=2^(*C/τ*)^ (6, 13, 40).

To further confirm the C period data obtained by qPCR method, we used a second method, the DNA increment method as described by Churchward and Bremer (41) and Bipatnath et al. (42), to measure the C period. This method is based on measuring the DNA increment after blocking DNA initiation of exponentially growing E. coli cells by the addition of 300 µg/ml chloramphenicol or 200 µg/ml rifampin (runoff experiments). The change in relative DNA amount after DNA initiation blockage corresponds to origins (*ori*)/genome equivalent.

The total DNA content per OD_600_ was measured by the diphenylamine colorimetric method, as detailed by Basan et al. (12). DNA content per cell was obtained through measuring total DNA amount per OD and cell number per OD through plating and a bacterial counting chamber by using microscopy. The origins (*ori*)/cell was obtained by multiplying genome equivalents per cell by calculating the *ori*-to-genome equivalent ratio. The D period is calculated based on the equation *ori/cell*= 2^(C+D)/τ^ (6, 13, 40).

The average initiation timing of the population was obtained using the equation t_i_ = N_oc × τ- (C+D), where N_oc is the number of overlapping cell cycles, equal to ceiling [(C+D)/τ]. Ceiling means the smallest integer that is larger than a specific value. The average initiation age, α (0 < α < 1) equals t_i_/τ (6, 9).

### Measurement of cellular DnaA abundance.

The level of DnaA protein was measured by quantitative mass spectrometry using the ^15^N-labeling method, as described by Hui et al. (43). The samples were analyzed on an AB Sciex TripleTOF 5600 system (AB Sciex, Framingham, MA). For the ^15^N reference cell sample, we used the cell culture growing exponentially in MOPS glucose medium (with ^15^NH_4_Cl as the nitrogen sources). Three hundred micrograms of the ^15^N reference cell sample (or labeled cell sample) was mixed with 300 μg of each of the ^14^N cell samples (or nonlabeled cell samples) growing in LB medium supplemented with different levels of sodium chloride. The mixed sample was then subject to trichloroacetic acid (TCA) and trypsin digestion, as detailed by Hui et al. (43). The mass spectrometry experiments and raw mass spectrum data files were processed similarly to those in a study by Hui et al. (43) to obtain the ratio of the ^14^N-nonlabeled to ^15^N-labeled peaks for each individual detected peptide, which gives the information of the relative abundance of related protein of each condition. For the DnaA protein, the relative protein abundance data were obtained as a ratio by taking the median of the ratios of its peptides. To guarantee that the ratio data are solid, only those DnaA data with at least three peptides detected were used. In addition, for the ratio data, if one or more of its quartiles lie outside the 50% range of its median, we treat the data as poor quality data and do not use it.

### Measurement of cellular dry mass/OD_600_.

The measurement of cellular dry mass is performed as the same as described by Basan et al. (12). Briefly, 200 ml to 250 ml cell culture was grown to exponential phase (OD_600_, ∼0.4) and was collected by centrifugation. The supernatant was completely and carefully removed. The cell pellets were further suspended in 2 ml double-distilled water (ddH_2_O), transferred to aluminum pans, and baked overnight under 70°C to reach constant weight, which corresponds to the dry mass. The exact OD_600_ value was recorded at the beginning and further calibrated with the cell culture lost (OD_600_) in each step to obtain the accurate OD_600_ value of the cell pellet determined for dry mass.
