# Supplementary figures and images for: High Osmolarity Modulates Bacterial Cell Size through Reducing Initiation Volume in Escherichia coli
Source: mSphere. 2018 Oct 24;3(5):e00430-18. doi: 10.1128/mSphere.00430-18 (PMC6200984; doi:10.1128/mSphere.00430-18)

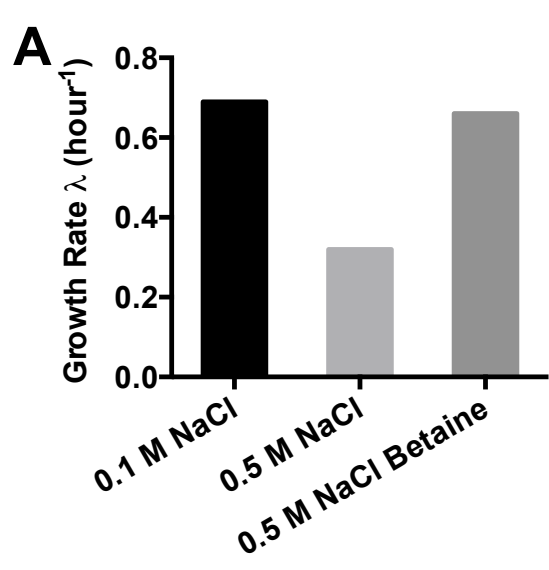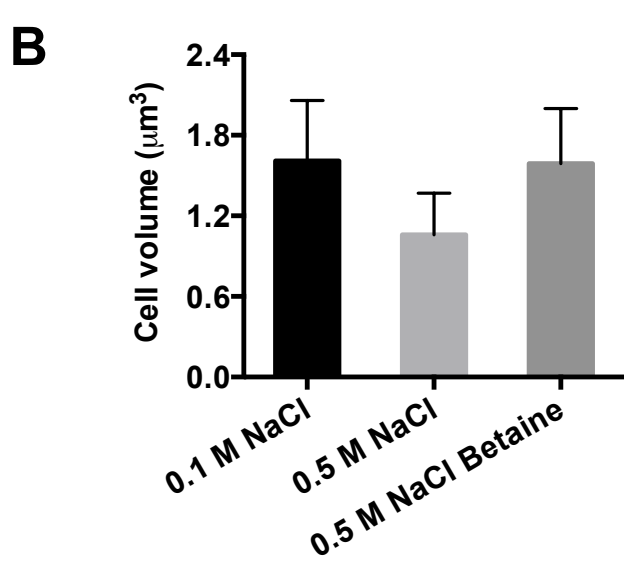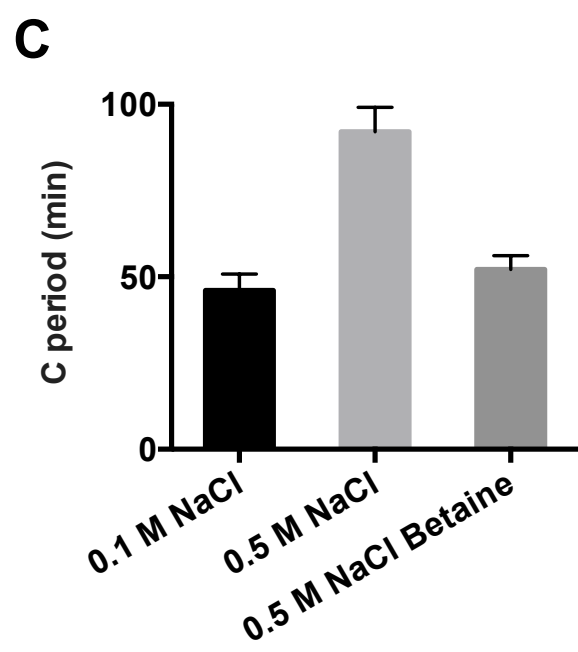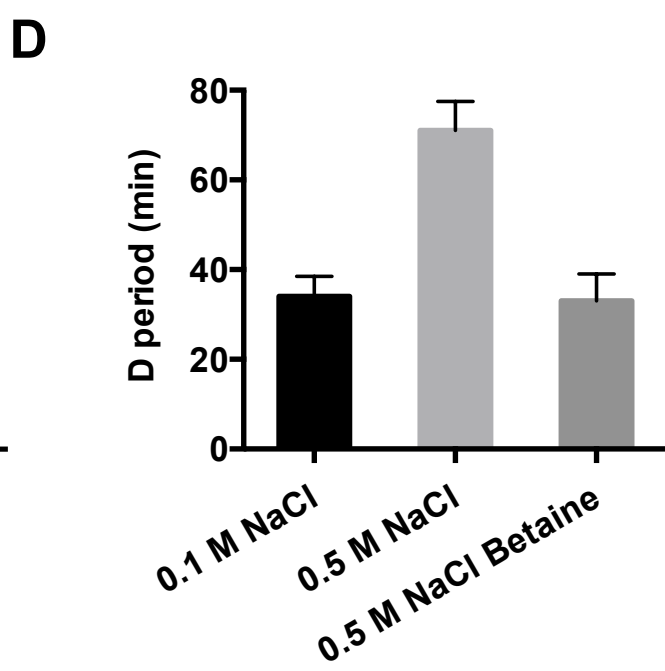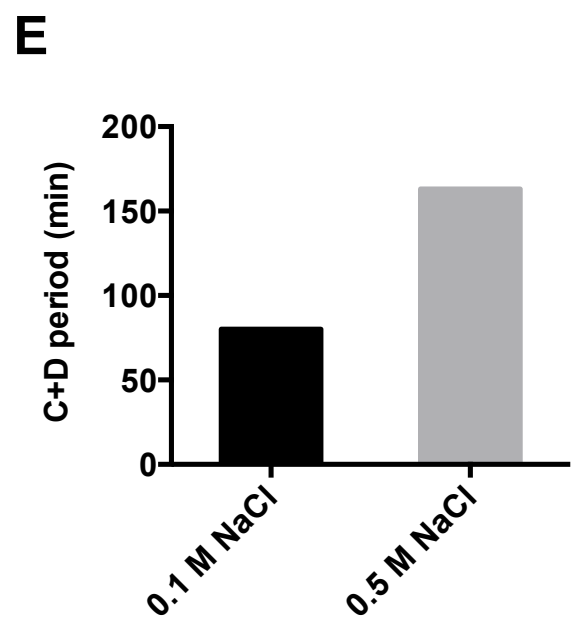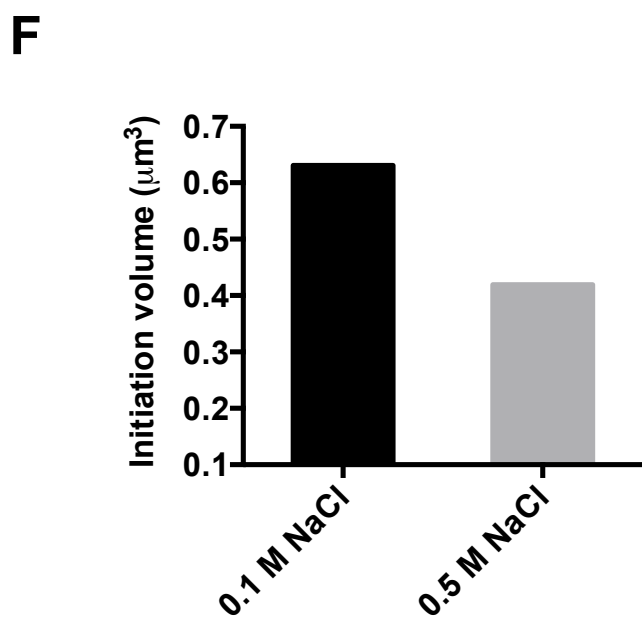

Supplement: FIG S1 [file sph005182665sf1.pdf]

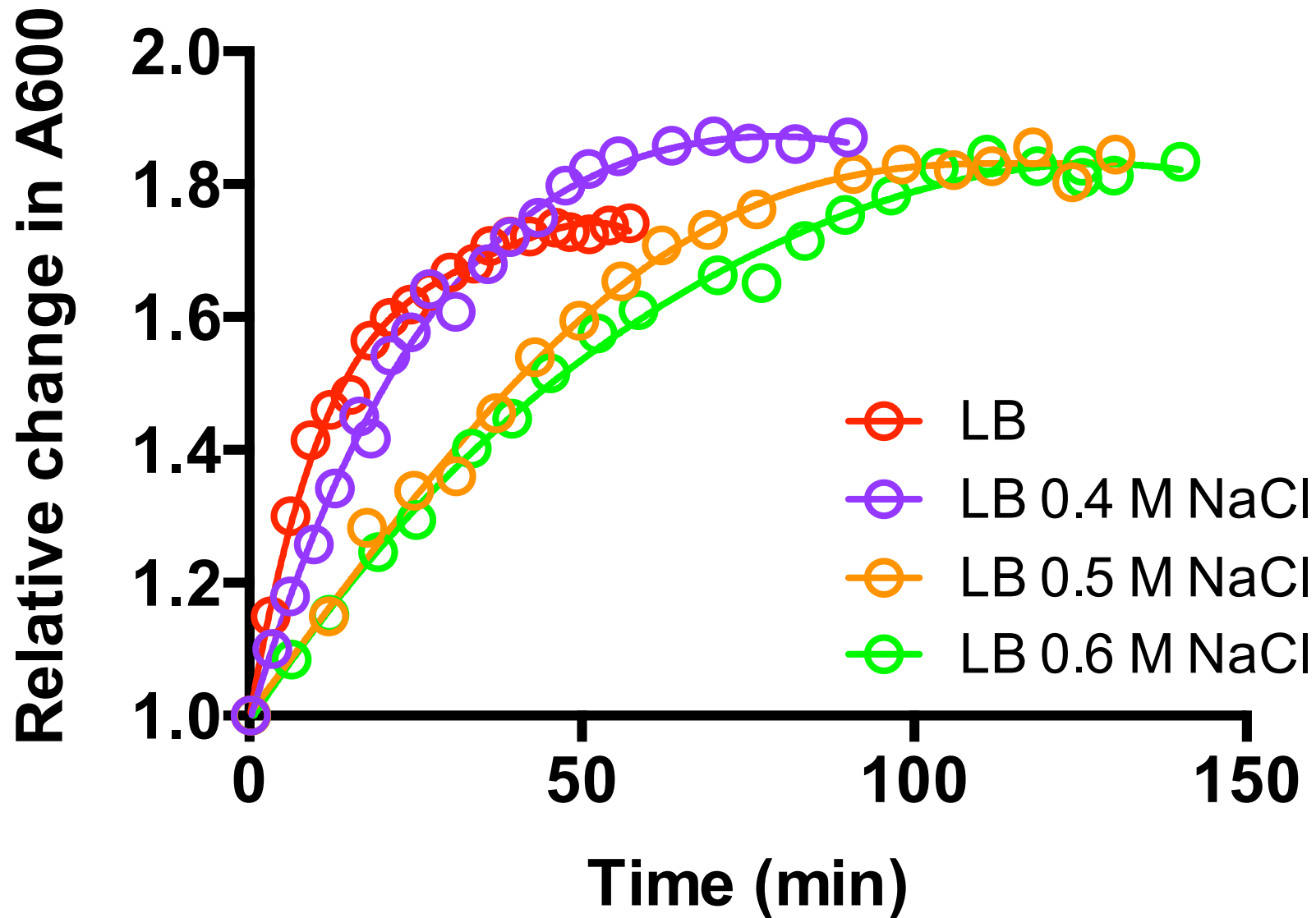

Supplement: FIG S2 [file sph005182665sf2.pdf]

**A**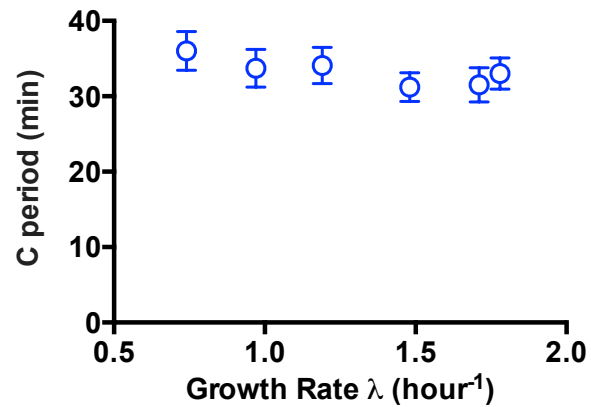**B**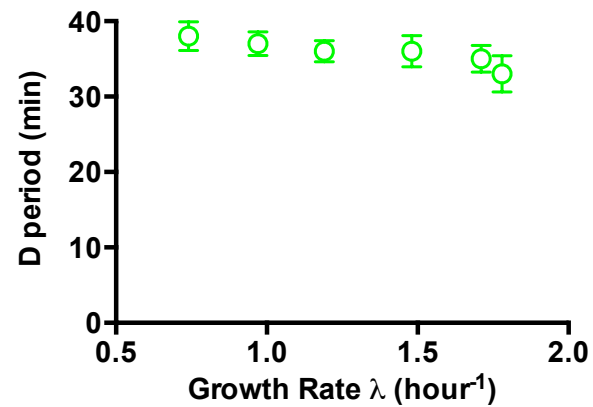**C**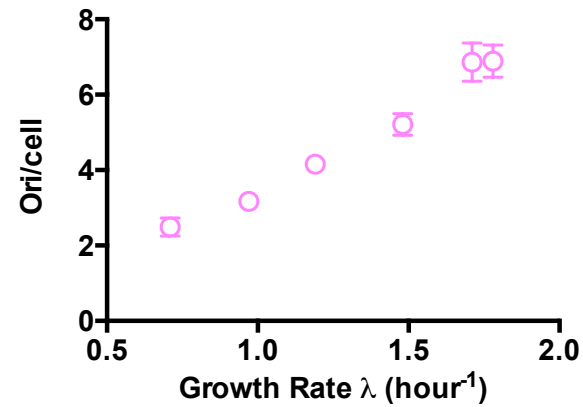**D**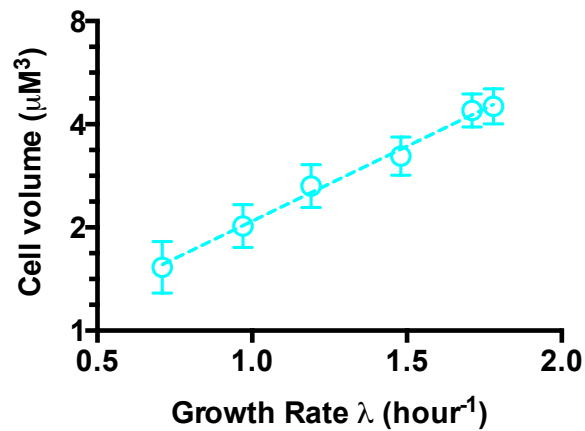**E**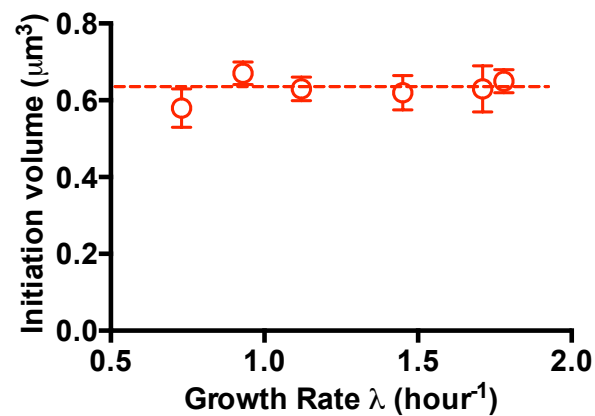

Supplement: FIG S3 [file sph005182665sf3.pdf]

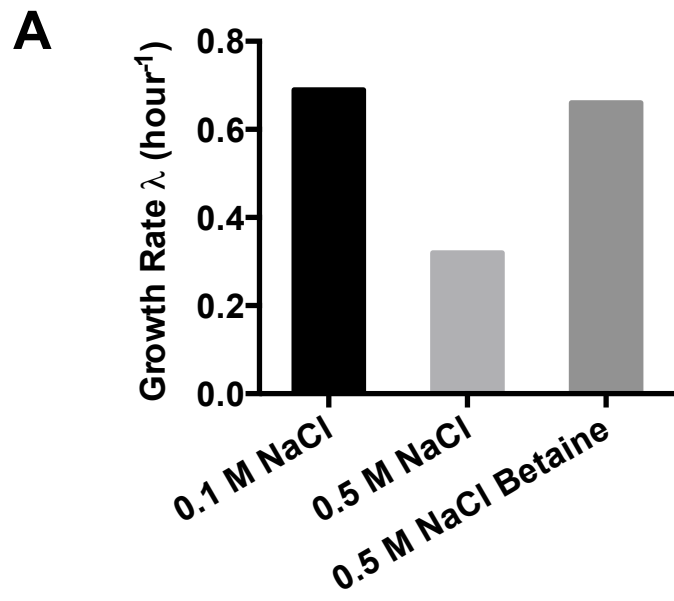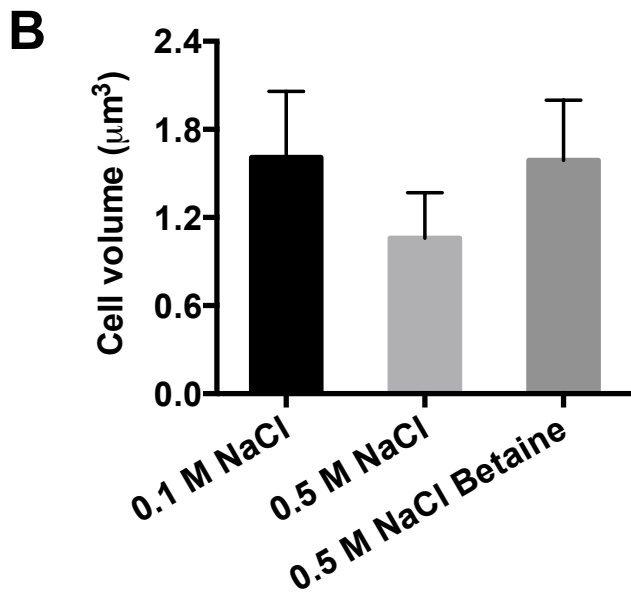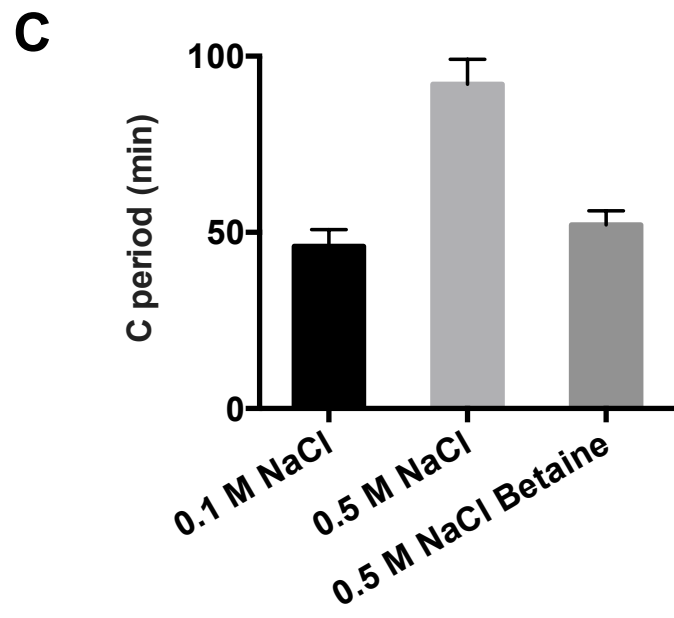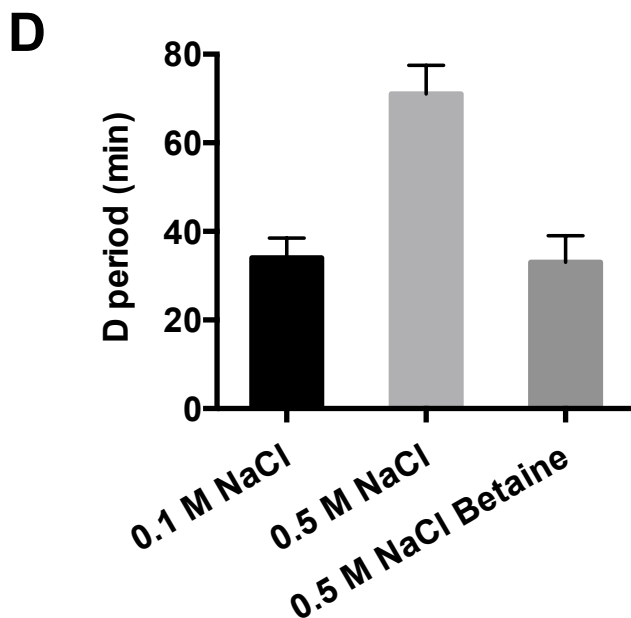

Supplement: FIG S4 [file sph005182665sf4.pdf]

**A**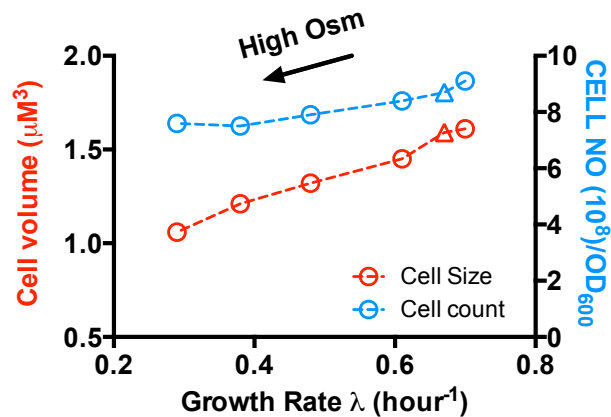**B**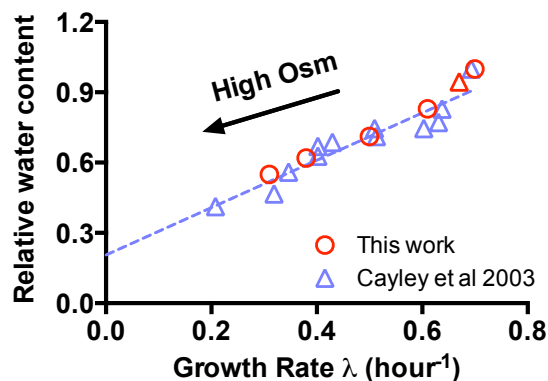**C**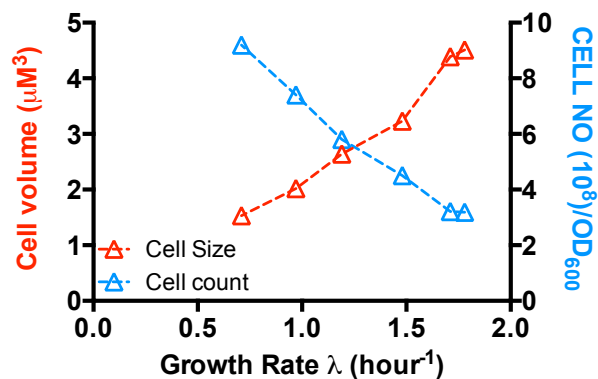**D**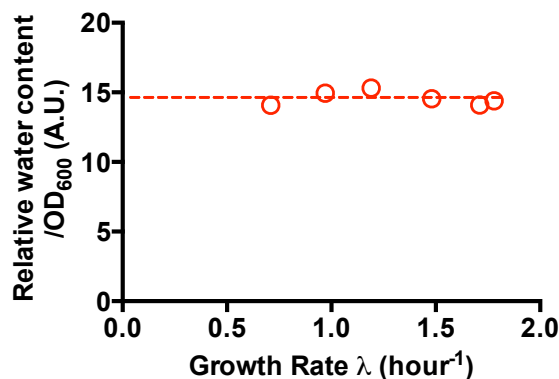**E**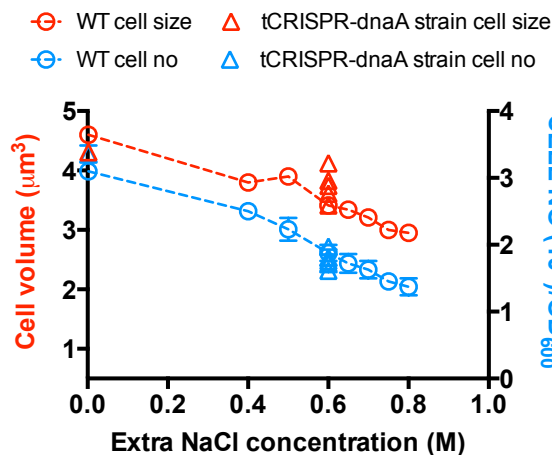**F**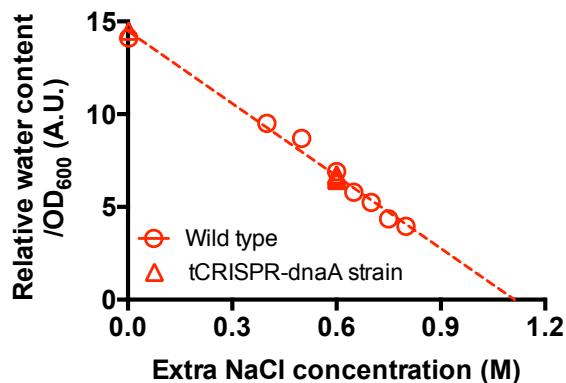

Supplement: FIG S5 [file sph005182665sf5.pdf]

**A**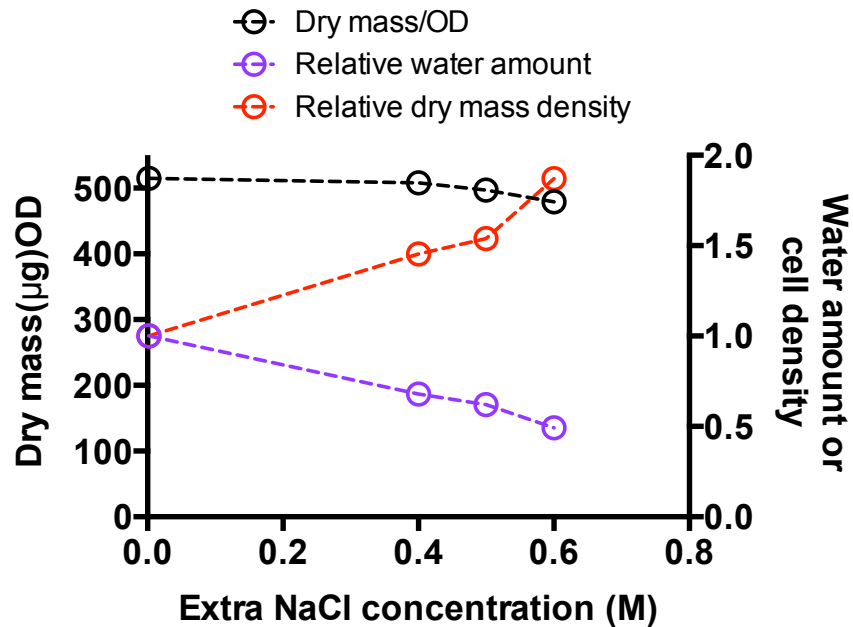**B**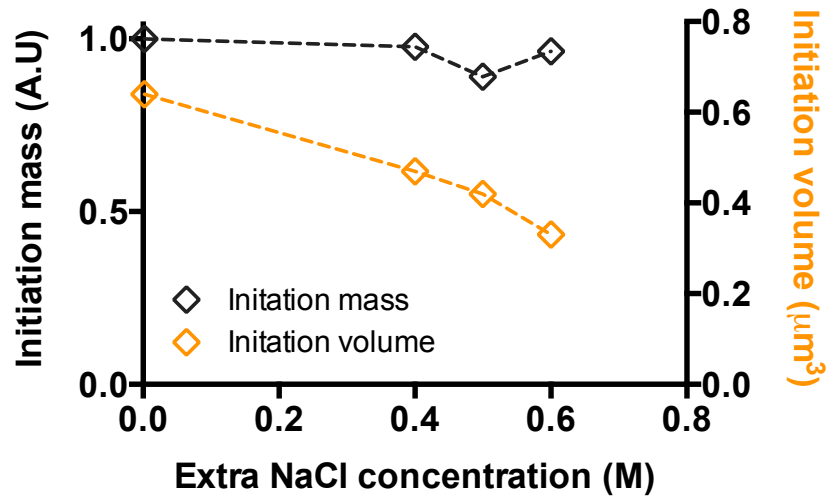

Supplement: FIG S6 [file sph005182665sf6.pdf]
